# Risk Factors and Outcomes of Early-Onset Sepsis in Very Preterm Infants: Results from the Thai Neonatal Registry (ThaiNy)

**DOI:** 10.3390/antibiotics15070674

**Published:** 2026-07-09

**Authors:** Junya Jirapradittha, Dissajee Lumbiganon, Anucha Thatrimontrichai, Buranee Swatesutipun, Kanmalee Jenjarat, Kullapat Srisomsap, Mananya Sukthong, Manunya Apiwattanaporn, Napas Sripirom, Nawamon Soontaravarapas, Nichanan Sahakaro, Ploypailin Preechawetchakul, Pornpimon Janyoungsak, Pornpimon Rojanakarin, Shanika Kosarat, Sudarat Sirichaipornsak, Sudatip Kositamongkol, Suparat Tipprasert, Tanin Pirunnet, Thaninee Chitsinchayakul, Walaiporn Bowornkitiwong, Chatchay Prempunpong

**Affiliations:** 1 Department of Pediatrics, Faculty of Medicine, Khon Kaen University, Khon Kaen 40002, Thailand; jjunya@kku.ac.th; 2 Department of Pediatrics, Faculty of Medicine, Navamindradhiraj University, Bangkok 10300, Thailand; dissajee@nmu.ac.th; 3 Department of Pediatrics, Faculty of Medicine, Prince of Songkla University, Songkhla 90110, Thailand; 4 Department of Pediatrics, Ramathibodi Hospital, Faculty of Medicine, Mahidol University, Bangkok 10400, Thailand; buranee.swa@mahidol.ac.th (B.S.); chatchay.pre@mahidol.ac.th (C.P.); 5 Nakornping Hospital, Chiang Mai 50180, Thailand; kanmalee.je@cpird.in.th; 6 Police General Hospital, Bangkok 10330, Thailand; kullapat.sr@police.go.th; 7 Buddhachinaraj Phitsanulok Hospital, Phitsanulok 65000, Thailand; mananya.bud@cpird.in.th; 8 Udonthani Hospital, Udon Thani 41000, Thailand; manunya.udh@cpird.in.th; 9 Bhumibol Adulyadej Hospital, Bangkok 10220, Thailand; napas.s@chula.ac.th; 10 Lampang Hospital, Lampang 52000, Thailand; ff_na10@hotmail.com; 11 Nopparat Rajathanee Hospital, Bangkok 10230, Thailand; jazznich@gmail.com; 12 Hatyai Hospital, Songkhla 90110, Thailand; ploypailin.pree@cpird.in.th; 13 Department of Pediatrics, Faculty of Medicine, Srinakharinwirot University, Nakhon Nayok 26120, Thailand; pornpimonj@g.swu.ac.th; 14 Sunpasitthiprasong Hospital, Ubon Ratchathani 34000, Thailand; p.rojanakarin@gmail.com; 15 Department of Pediatrics, Faculty of Medicine, Chiang Mai University, Chiang Mai 50200, Thailand; shanika.k@cmu.ac.th; 16 Sakon Nakhon Hospital, Sakon Nakhon 47000, Thailand; noinanewborn@gmail.com; 17 Department of Pediatrics, Faculty of Medicine, Thammasat University, Pathumthani 12120, Thailand; ksudatip@tu.ac.th; 18 Roi Et Hospital, Roi Et 45000, Thailand; suparattipprasert@gmail.com; 19 Department of Pediatrics, Phramongkutklao Hospital and Phramongkutklao College of Medicine, Bangkok 10400, Thailand; tanin.pi@pcm.ac.th; 20 Department of Pediatrics, Faculty of Medicine, Chulalongkorn University, Bangkok 10330, Thailand; thaninee.c@chula.ac.th; 21 Department of Pediatrics, Siriraj Hospital, Faculty of Medicine, Mahidol University, Bangkok 10700, Thailand; valaiporn.bow@mahidol.ac.th

**Keywords:** *Acinetobacter baumannii*, antimicrobial drug resistance, antimicrobial stewardship, bacteremia, bacterial meningitis, bacterial multidrug resistance, neonatal sepsis

## Abstract

Objective: To determine the incidence, causative pathogens, risk factors, and clinical outcomes of early-onset sepsis (EOS) in very preterm (gestational age < 32 weeks) and very low birth weight (birth weight < 1500 g) neonates. Methods: This retrospective study analyzed data from the Thai Neonatal Network registry (2019–2024; total 22 neonatal units). EOS was defined as bacteremia or bacterial meningitis within 72 h of birth. Results: Among 792 neonates (median [interquartile range] gestational age, 28 [26–29] weeks; median [interquartile range] birth weight, 960 [779–1190] g), the incidence of EOS was 4.0% (*n* = 32). Predominant pathogens were *Escherichia coli* (*n* = 10) and *Acinetobacter baumannii* (*n* = 9). Antibiograms showed high non-susceptibility for *E. coli* and *A. baumannii* to gentamicin (44.4% and 83.3%, respectively) and cefotaxime (37.5% and 75.0%, respectively). Although *E. coli* remained susceptible to amikacin and meropenem (100%), *A. baumannii* exhibited extensive resistance (66.7% and 83.3%, respectively). Multivariable analysis identified chorioamnionitis (adjusted odds ratio [aOR] 1.12; 95% confidence interval [CI]: 1.07–1.18) and 5 min Apgar score < 7 (aOR 1.05; 95% CI: 1.02–1.18) as significant risk factors. EOS was independently associated with increased early neonatal mortality within 7 days of life (aOR, 1.26; 95% CI: 1.16–1.36). Conclusions: The incidence of EOS is high among very preterm infants in Thailand and significantly increases mortality rates. The prevalence of multidrug-resistant *A. baumannii* in neonatal EOS is a critical concern that limits the efficacy of empirical treatments. These findings highlight the urgent need for targeted interventions and enhanced antimicrobial stewardship to improve neonatal survival rates.

## 1. Introduction

Neonatal early-onset sepsis (EOS) is a critical condition associated with significant mortality, long-term neurodevelopmental sequelae, and cognitive impairment [1,2]. However, the incidence, leading pathogen and susceptibility, and clinical outcomes of EOS vary across different neonatal settings and are influenced by local epidemiology, environmental colonization in the maternal unit, delivery room and neonatal unit, and the incidence of antimicrobial resistance. Previous studies have reported a global incidence of neonatal EOS of 0.5–1 per 1000 live births [3,4,5,6,7,8,9,10]. Although the case fatality rate (CFR) in the United States ranges from 11.1% to 16.2% [4,6], it increases sharply to 35.0% in very low birth weight (VLBW; birth weight [BW] < 1500 g) neonates [11]. In the United States and Europe, Group B Streptococcus (GBS) remains the most prevalent pathogen of EOS in both full-term and VLBW neonates. However, the incidence of *Escherichia coli* has increased in regions utilizing universal intrapartum antibiotic prophylaxis (IAP) for GBS [11]. GBS remains the primary causative pathogen in Western countries [3,6,7,8], whereas GBS EOS is rare in Southeast Asia [12,13]. Despite these observations, the epidemiology of EOS in Southeast Asia remains under-characterized and has a high rate of antimicrobial resistance, particularly among very preterm and VLBW infants.

Southeast and South Asia have high rates of colonization and infection by *Acinetobacter baumannii* [14], particularly occurring in neonatal bacteremia [15,16,17], meningitis [17,18], and ventilator-associated pneumonia [19,20]. In a neonatal intensive care unit (NICU) in Thailand [21] and three NICUs in India [17], the most common causative organisms of EOS were *A. baumannii* (19/60 [32%] and 157/580 [27%], respectively) and carbapenem-resistant *A. baumannii* (CRAB; 12/19 [63%] in EOS [21] and 173/215 [80%] in both EOS and late-onset sepsis [17], respectively). Furthermore, the emergence of CRAB presents a significant challenge due to inadequate antimicrobial coverage.

To gain a comprehensive understanding of the incidence, predominant pathogens, and clinical outcomes of EOS among VLBW infants within the Thai Neonatal Registry (ThaiNy), we conducted a cohort study focusing on very preterm (gestational age [GA] < 32 weeks) and VLBW neonates. The primary objective of this study was to determine the incidence of EOS while identifying its causative organisms, risk factors, and associated clinical outcomes to inform infection prevention and control strategies. A specialized subgroup, the Thai Neonatal Network Sepsis Working Group, was established for this study. This collaborative effort included 22 university-affiliated and public hospitals across Thailand dedicated to the collection of clinical characteristics, including comprehensive microbiological profiles, antimicrobial susceptibility patterns, and clinical outcomes for each EOS case.

## 2. Results

Over the 4.5-year study period, 995 VLBW or very preterm neonates were screened. After excluding neonates with BW ≥ 1500 g (*n* = 73), GA ≥ 32 weeks (*n* = 83), congenital anomalies (*n* = 7), and incomplete medical records (*n* = 40), a final cohort of 792 neonates was included in the analysis. The median GA and BW were 28 weeks (interquartile range [IQR], 26–29; range, 20–31) and 960 g (IQR, 779–1190; range, 350–1496), respectively. The cumulative incidence of EOS was 4.0% (32/792 neonates). Among these 32 neonates, 32 distinct episodes involving 38 isolated organisms were recorded. A comprehensive comparison of the baseline characteristics and clinical outcomes of neonates with and without EOS is presented in Table 1 and Table 2, respectively.

The 38 isolates comprised a diverse range of pathogens (Table 3). Gram-negative bacteria were predominant and included *E. coli* (*n* = 10), *A. baumannii* (*n* = 9), *Haemophilus influenzae* (*n* = 2), *Campylobacter fetus* (*n* = 1), *Klebsiella pneumoniae* (*n* = 1), *Moraxella* species (*n* = 1), and *Pseudomonas aeruginosa* (*n* = 1). Gram-positive isolates included *Listeria monocytogenes* (*n* = 1), Staphylococcus *aureus* (*n* = 1), and *Streptococcus agalactiae* or GBS (*n* = 1). Ten isolates were identified as common commensal organisms: Coagulase-negative staphylococci (CoNS) or *Staphylococcus epidermidis* (*n* = 7), *Micrococcus* species (*n* = 1), *Staphylococcus capitis* (*n* = 1), and *Staphylococcus hominis* (*n* = 1). These isolates were considered causative of EOS based on clinical criteria: nine cases presented with clinical sepsis requiring ≥ 5 days of antibiotic therapy, and one case of CoNS was confirmed by two positive blood cultures within 24 h. Among Gram-negative pathogens, the rates of non-susceptibility to gentamicin, cefotaxime, meropenem, and amikacin were 68.2%, 50.0%, 33.3%, and 27.8%, respectively. All five neonates with meropenem-resistant *A. baumannii* occurred within the 48-to-72 h window after birth. Regarding Gram-positive pathogens, all *S. epidermidis* isolates showed methicillin resistance (100%), whereas no methicillin-resistant *S. aureus* was detected. Detailed antimicrobial susceptibility profiles are provided in Table 3.

The case fatality rates (CFR) for each organism are detailed in Table 3. Notably, 100% mortality rates were observed in neonates with sepsis caused by *H. influenzae* (2/2), *K. pneumoniae* (1/1), *P. aeruginosa* (1/1), and *S. capitis* (1/1). In contrast, all infants with EOS attributed to *C. fetus*, *L. monocytogenes*, *Micrococcus* species, *Moraxella* species, *S. aureus*, *S. hominis*, and GBS survived until discharge. The CFRs of *A. baumannii*, *E. coli*, and CoNS were 55.6%, 30.0%, and 28.6%, respectively.

Variables identified as potential risk factors in the univariable analysis (*p* < 0.20) were entered into a multivariable logistic regression model (Table 4). In the final adjusted model, the EOS group was significantly more likely to have a history of clinical chorioamnionitis (adjusted odds ratio [aOR] 1.12; 95% confidence interval [CI]: 1.07–1.18; *p* < 0.001) and 5 min Apgar score < 7 (aOR 1.05; 95% CI: 1.02–1.08; *p* = 0.002) compared to the non-EOS group.

The overall early neonatal mortality (ENM) rate, defined as death within 7 days of birth, was 6.19% (49/792) in the very preterm cohort. Maternal and neonatal factors associated with ENM in the univariable analysis are presented in Table 5.

Variables with a *p*-value < 0.20 in the univariable analysis were included in a multivariable logistic regression model (Table 6). In the final adjusted model, EOS remained independently associated with a significant increase in the odds of ENM (aOR 1.26; 95% CI: 1.16–1.36; *p* < 0.001). Additional independent predictors of mortality included lower GA, delivery at public hospitals, small for gestational age (SGA) status, and the presence of persistent pulmonary hypertension of the newborn (PPHN).

## 3. Discussion

Based on data from the ThaiNy registry from 2019 to 2024, the incidence of EOS in VLBW neonates was 4.0% (32/792). When excluding nine commensal organisms (identified from a single positive specimen), the incidence remained significant at 2.9% (23/792). The microbiological landscape was dominated by Gram-negative bacteria, specifically *E. coli* and *A. baumannii*. The rate of non-susceptibility to gentamicin was as high as 68.2%. The highest CFR was observed in *A. baumannii* sepsis, which also exhibited a critical resistance of 83.3% to meropenem. In contrast, the incidence of GBS EOS remained low despite the lack of routine GBS screening or universal IAP in Thailand. Multivariable analysis identified clinical chorioamnionitis and 5 min Apgar score < 7 as independent risk factors for EOS. Furthermore, the presence of EOS was significantly associated with increased ENM, highlighting its impact on survival in this vulnerable population.

### 3.1. Incidence of EOS

The reported incidence of EOS varies significantly according to geographic region, resource availability, study scale (single center vs. network), and the specific timeframe of the study. In our cohort of VLBW infants, the incidence of 4.0% (or 2.9% when excluding commensals) is generally higher than that reported in Europe (1–3%), North America, Australia, and New Zealand (1–2%) (Table 7). The incidence of EOS in Asia is highly variable. Previous studies have reported rates of <1% in Taiwan [22,23], and between 1% and 5% in Malaysia [24], Israel [25], South Korea [26,27], and Thailand [21]. It is important to note that in studies with smaller cohorts of VLBW infants, the reported incidence of EOS can appear disproportionately high. For instance, research from China and Indonesia has documented EOS rates as high as 10% [28,29].

These disparities are often compounded by the inconsistent definitions of EOS in the literature. For example, while many studies, including ours, define preterm EOS as the isolation of a pathogenic bacterial species from a blood or cerebrospinal fluid (CSF) culture obtained within 72 h of birth, others may use a 48 h or 7-day cutoff. Such variations in surveillance criteria and the inclusion of commensal organisms make direct global comparisons challenging [30,31]. EOS was defined as the isolation of pathogenic species from the blood or CSF within 72 h of birth and antibiotic treatment for at least 5 days or until death [25,32,33]. True infections by commensal species were identified by comparing growth in the two specimens [30] or from two or more blood specimens collected on separate occasions [34]. EOS was defined as sepsis within the first 2 [7], 3 [22,24,25,28,30,31,32,33,35,36,37,38] or 7 [23,26] days of life.

**Table 7:** Comparison of incidence and case fatality rates of early-onset sepsis in very low birth weight infants: Regional and international perspectives.

Country or Network [Reference]	Years of Collection	Incidence, *n* (%)	Case Fatality Rate, *n* (%)
The Israel Neonatal Network [25]	1995–2005	383/15,839 (2.42%)	150/383 (39.2%)
Taiwan [23]	2001–2006	2/590 (0.34%)	
Taiwan [22]	2005–2009	10/1042 (0.96%)	4/10 (40.0%)
South Korea [27]	2013–2015	212/4191 (5.06%)	
The Korean Neonatal Network [26]	2013–2014	85/2386 (3.56%)	29/85 (34.1%)
China [33]	2015–2018	143/10,848 (1.32%)	38/143 (26.6%)
China [28]	2019–2020	37/369 (10.03%)	
China [39]	2017–2023	Not Available (0.83%)	
The Malaysian National Neonatal Registry [24]	2010	61/3880 (1.57%)	31/61 (50.8%)
Indonesia [29]	2012–2013	27/258 (10.47%)	
Thailand [21]	2004–2023	23/1223 (1.88%)	13/23 (56.5%)
The Thai Neonatal Registry [This study]	2019–2024	32/792 (4.0%)	13/32 (40.6%)
The European Neonatal Network database [36]	2006–2009	391/14,719 (2.65%)	
Germany [2]	2008–2011	3/342 (0.88%)	
The German Neonatal Network [40]	2009–2017	168/14,855 (1.13%)	21/168 (12.2%)
Poland [37]	2009	25/910 (2.75%)	10/25 (40.0%)
Italy [41]	2011–2012 and 2016–2017	7/230 (3.04%)	
The NICHD [42]	1991–1993	147/7606 (1.93%)	38/147 (25.8%)
The NICHD [43]	1998–2000	84/5447 (1.54%)	31/84 (36.9%)
The NICHD [44]	2002–2003	102/5999 (1.70%)	36/102 (35.3%)
The NICHD [32]	2015–2017	88/6322 (1.39%)	30/88 (34.1%)
The Vermont Oxford Network [45]	2018–2019	1139/84,333 (1.35%)	
United States [31]	1990–2015	109/5313 (2.05%)	
North America by the Pediatrix Medical Group [46]	1997–2010	1032/108,000 (0.95%)	
The Canadian Neonatal Network [38]	2005–2007	94/7141 (1.32%)	
The Australian and New Zealand Neonatal Network [38]	2005–2007	156/9995 (1.56%)	

NICHD, National Institute of Child Health and Human Development.

### 3.2. Leading Pathogens

The causative pathogens of EOS vary significantly by geographical region. In the United States, *E. coli* and GBS are the predominant organisms. Since the introduction of the Centers for Disease Control and Prevention (CDC) guidelines for the prevention of perinatal GBS in 1996, which mandated universal screening and IAP for GBS-positive or unknown status, there has been a marked shift in the microbiological landscape [47]. Specifically, the rate of GBS-associated EOS has steadily declined, dropping from 30.6% (45/147) in 1991–1993 to 11.5% (9/78) in 2015–2017 [32,42,43,44]. Conversely, the relative proportion of *E. coli* EOS increased significantly over the same period, rising from 16.3% (24/147) in 1991–1993 to 57.7% (45/78) in 2015–2017. This trend suggests that although IAP has been highly effective in mitigating GBS, it may inadvertently contribute to the prominence of Gram-negative pathogens in the EOS profile of VLBW infants.

Similarly, data from major European networks have highlighted the dominance of Gram-negative organisms and commensals in very preterm EOS. In the European Neonatal Network (*n* = 393 isolates), *E. coli* (25.0%) and CoNS (22.5%) were the most prevalent pathogens, followed by GBS (12.8%), *Enterococcus* spp. (6.6%), *S. aureus* (4.1%), and *L. monocytogenes* (4.1%) [36]. A similar trend was observed in the German Neonatal Network (*n* = 168 isolates), where *E. coli* (34.6%) and CoNS (23.9%) remained the leading causes of EOS, with *Enterobacter* spp. (4.2%) appearing less frequently [40]. This microbiological profile is consistent with findings from the Israel Neonatal Network, where the most common isolates were *E. coli* (26.8%), CoNS (17.2%), and GBS (9.4%) [25]. In contrast, a Polish study reported a different hierarchy, with GBS (20.0%; 5/25) as the primary organism, followed by CoNS (16.0%; 4/25) and *E. coli* (12.0%; 3/25) [37]. These variations underscore the heterogeneity of EOS pathogens, even within Europe, likely reflecting localized differences in IAP protocols and NICU environments.

The microbiological profiles of EOS in East Asia showed significant regional variation. In China and Taiwan, *E. coli* (24.5–40.0%) and *K. pneumoniae* (18.4–20.0%) were identified as primary pathogens in VLBW cohorts [22,35]. However, other Chinese studies have reported a different hierarchy, with *Enterococcus faecalis* (24.0%), *Streptococcus* spp. (21.0%), and *L. monocytogenes* (15.0%) predominating [28]. A large-scale network study in South Korea identified CoNS as the leading isolate (56.5%), followed by *E. coli* (11.8%) and *S. aureus* (10.6%), with *Acinetobacter* species accounting for only a small fraction (5.9%) of cases [26].

In South Asia, specifically within the Indian neonatal population, the spectrum of causative pathogens of EOS in VLBW infants exhibits significant regional variation compared to global trends. In an Indian NICU in 2018, the most common pathogens were *S. aureus* (12/40, 30%), *Klebsiella* spp. (7/40, 18%), *Pseudomonas* spp. (7/40, 18%), and *Acinetobacter* spp. (5/40, 13%) [48]. Whereas in another Indian NICU from 2019 to 2021, the bacterial pathogens were *E. coli* (21/54, 39%), *Klebsiella* (13/54, 24%), *S. aureus* (6/54, 11%), *Legionella* (5/54, 9%), *Pseudomonas* (5/54, 9%), *Burkholderia* (3/54, 6%), and *Acinetobacter* (1/54, 2%) [49].

In Southeast Asia, the pathogen distribution appears to shift toward a higher prevalence of GBS and emerging Gram-negative threats. Data from Malaysia indicated that GBS (26.2%) and *Acinetobacter* species (16.4%) were the most frequent isolates in VLBW infants [24]. Our study in Thailand further underscores this regional trend, with Gram-negative bacteria as the dominant microorganism. The leading pathogens were *E. coli* (26.3%), *A. baumannii* (23.7%), and CoNS (18.4%). Notably, the prevalence of *A. baumannii* in our cohort was higher than that reported in Malaysia and significantly exceeded the rates observed in East Asian and Western networks. The high prevalence of *Acinetobacter*, coupled with its extensive resistance profile, represents a unique and critical challenge for empirical antibiotic selection in the Thai neonatal population.

In summary, the distribution of causative pathogens of EOS among VLBW infants is highly dependent on the geographical region, study period, and cohort size. Distinct microbiological profiles emerge across different continents: the United States (*E. coli* and GBS), Europe (*E. coli* and CoNS), East Asia (*E. coli* and *K. pneumoniae*), South Asia (*S. aureus* and *Klebsiella*), and Southeast Asia (GBS, *E. coli*, and *A. baumannii*). Notably, *A. baumannii* appears to be a regional challenge in Asia, as reported in Malaysia [24], South Korea [26], India [48,49], and in this study. In contrast, this pathogen was notably absent or not reported in major surveillance networks in the United States [31,32] and Germany [40], and in certain studies in China [35]. These disparities highlight the shifting epidemiology of neonatal sepsis and underscore the urgent need for region-specific empirical antibiotic guidelines, rather than a “one-size-fits-all” global approach.

### 3.3. Risk Factors

From a meta-analysis [50], the risk factors for EOS in full term and preterm neonates were vaginal examination ≥ 3 times (OR = 7.95, 95% CI: 4.04–15.64), chorioamnionitis (OR = 4.58, 95% CI: 2.61–8.05), meconium-stained amniotic fluid (OR = 4.51, 95% CI: 2.31–8.81), VLBW infant (OR = 3.79, 95% CI: 2.14–6.73), maternal urinary or reproductive tract infection (OR = 3.61, 95% CI: 2.14–6.11), perinatal fever (OR = 3.59, 95% CI: 2.25–5.71), perinatal asphyxia or intrauterine distress (OR = 3.00, 95% CI: 2.18–4.13), premature rupture of membranes (OR = 2.63, 95% CI: 2.09–3.30), GBS colonization in pregnant women (OR = 2.13, 95% CI: 1.48–3.05), and lower GA (OR = 1.31, 95% CI: 1.18–1.44).

In VLBW infants, the risk factors for EOS were lower GA or BW [2,24,25,26,36,40], chorioamnionitis or intra-amniotic infection [25,40], spontaneous delivery [40], meconium-stained amniotic fluid [35], antenatal steroids exposure [36], single gestation [36], low 5 min Apgar score [36], delivery room resuscitation [25,36], endotracheal tube ventilation at birth [24], and pneumonia [24]. In the present study, the risk factors for EOS were chorioamnionitis and 5 min Apgar score < 7. Although neonates with lower GA or BW had an increased risk of EOS in the univariable analysis, there was no significant difference in the multivariable analysis.

In the Malaysian National Neonatal Registry [24], compared with NICUs reporting no EOS (*n* = 14), NICUs reporting EOS (*n* = 20) had significantly higher patient loads (total live births, admissions, VLBW infants, and outborns); more mothers with a history of abortions, and antenatal steroids and intrapartum antibiotic use; more infants requiring resuscitation procedures at birth; and higher rates of surfactant therapy, pneumonia, and insertion of central venous catheters. In the ThaiNy registry, university-affiliated hospitals were associated with a higher rate of EOS than public hospitals.

### 3.4. Outcomes

The CFR or mortality rate at discharge of VLBW infants with EOS was significantly higher than that of infants without EOS in Malaysia [24], Israeli [25], German [40], and European [36] studies, and in this study, but there was no difference in a Korean study [26]. The CFR of EOS in this study (40%) was similar to that in studies in Taiwan [22], Poland [37], and Israel [25], but lower than that in Malaysia [24] and Thailand [21], and higher than that in Korea [26], Germany [40], and the National Institute of Child Health and Human Development (NICHD) [32] (Table 7). The pathogen with the highest CFR was *A. baumannii* (5/9 [55.6%]), which was not susceptible to gentamicin or meropenem (5/6 [83.3%]). *A. baumannii* in EOS was has been reported in EOS in Malaysia [24], Korea [26], and Thailand [21].

The ENM rate in this study (10/32 [31.2%]) was similar to that reported in the Israel Neonatal Network (109/383 [28.5%]) [25]. ENM was associated with the EOS (aOR 1.26, 95% CI 1.16–1.36, *p* < 0.001), GA, public hospitals, SGA, and PPHN. Similar to a previous study, the mortality rate of VLBW neonates was associated with SGA, PPHN, and lower GA or 5 min Apgar score [51]. Morbidities at discharge of EOS in VLBW infants include intraventricular hemorrhage [2,25,26,36], severe retinopathy of prematurity [25,36], and bronchopulmonary dysplasia [25]. In a German study [2], EOS among VLBW infants significantly impaired neurodevelopment at 2 years of corrected age.

### 3.5. Strengths and Limitations

A primary strength of this study is the characterization of *A. baumannii* as a significant EOS pathogen, reflecting a broader epidemiological trend in Southeast Asia, where high rates of colonization and infection have been documented [14]. The high prevalence of antimicrobial resistance, particularly CRAB, is a critical concern, as it often renders standard empirical antimicrobial therapy inadequate. Unlike many traditional EOS pathogens that are vertically transmitted from mother to neonate, *A. baumannii* is more frequently associated with horizontal transmission in healthcare settings. It colonizes water reservoirs and medical equipment, particularly ventilator circuits. Our findings are consistent with a previous surveillance study from a Thai NICU [21] that identified *A. baumannii* as the most prevalent EOS isolate among both full-term and preterm neonates. These results suggest that in the Thai context, the boundary between vertically transmitted “early-onset” sepsis and healthcare-associated infection may be increasingly blurred by environmental pathogens.

Nevertheless, our findings must be interpreted with caution, considering some limitations. First, the definition of EOS has been inconsistent. Discrepancies exist regarding the duration of onset, which ranges from 72 h [22,24,28,32,35,36] to 7 days [23,26], and the classification of commensal organisms. For instance, the NICHD Neonatal Research Network [32] requires antibiotic treatment for ≥5 days for a culture to be considered EOS, while the CDC’s National Healthcare Safety Network [34] and the Vermont Oxford Network [52] employ stricter criteria for commensals (e.g., CoNS), necessitating multiple positive cultures. Second, the study was affected by data constraints. Significant information on antimicrobial susceptibility was lacking, particularly for ampicillin, third-generation cephalosporins, and colistin. Furthermore, we lacked data on critical risk factors such as maternal GBS colonization status, environmental/NICU colonization, and antibiotic utilization ratio, which are vital for understanding the transmission of *A. baumannii*. Colonization by antimicrobial-resistant pathogens may occur due to frequent and prolonged broad-spectrum antimicrobial use [53]. Third, the specific risk factors driving *A. baumannii* or antimicrobial resistance were not fully identified because of the limited number of cases. However, a previous Thai study [21] suggested that neonates with EOS not susceptible to ampicillin and gentamicin were more likely to have a later onset (48–72 h of life) and require early endotracheal intubation. *A. baumannii* is traditionally a healthcare-associated and ventilator-associated pathogen that typically manifests after at least 48 h of hospitalization. However, EOS is usually defined as occurring within 72 h of birth. Similarly, in this study, all cases of meropenem-resistant *A. baumannii* infection emerged within the 48-to-72 h window after birth. Fourth, owing to the absence of reported long-term morbidities typically linked to late-onset sepsis, our analysis used ENM as the definitive clinical endpoint for EOS. Finally, geographical and sample size limitations may have affected generalization. Although this was a multicenter study, the number of patients with EOS was relatively small. Given that the incidence and pathogen profiles depend on the study period, regional epidemiology, and resource allocation, our results reflect Thailand’s unique landscape.

## 4. Materials and Methods

### 4.1. ThaiNy Database and Patient Domains

This multicenter, retrospective, observational study utilized data from ThaiNy, a multicenter prospective registry established in 2019 by the Thai Neonatal Society. Initially launched in 2019, the registry encompasses 28 Level III-IV neonatal care units [54] across private, public, and university hospitals in Thailand. The registry employs a secure web-based electronic data capture system with multiple layers of data protection. Each participating hospital designated trained personnel to be responsible for data entry and quality assurance. The personnel responsible for data entry received standardized training on registry protocols and data definitions. Neonates were assigned unique registry identification numbers to maintain confidentiality, with personal identifiers stored separately from the clinical data in encrypted databases. Access to the registry system was restricted through role-based authentication protocols, with audit trails maintained for all data entries and modifications. Although the data were derived from the registry, supplemental antibiograms were collected independently. After excluding some private and university hospitals owing to incomplete medical records, 22 NICUs were included in the final analysis.

Neonates with GA < 32 weeks or BW < 1500 g were eligible for inclusion. Patients were excluded if they met the following criteria: (1) BW ≥ 1500 g; (2) GA ≥ 32 weeks; (3) presence of congenital anomalies; and (4) incomplete medical record. Data were collected from October 2019 to June 2024 using a standardized case record form and subsequently entered into a secure database for analysis. Organism classification and antimicrobial susceptibility coding were performed according to predefined study definitions to ensure consistency across participating centers.

This study was conducted in accordance with the Declaration of Helsinki and was approved by the Thai Central Research Ethics Committee of all participating hospitals (COA-CREC 053/2019), Khon Kaen University (HE691344), and Prince of Songkla University (REC. 69–209–1–1). As this was a registry-based study using de-identified data collected as part of routine clinical care quality improvement initiatives, the requirement for informed consent was waived by the ethics committees in accordance with local regulations. However, informed consent had previously been obtained for participation in the Thai Neonatal Network registry. Data confidentiality and security protocols were strictly maintained throughout the study.

### 4.2. Definitions

In this study EOS was defined as the isolation of bacterial pathogens from blood or CSF within 72 h of birth [22,24,25,28,32,35,36,37,38]. Commensal species were considered true infections when comparing growth in two or more blood specimens [34] or one blood specimen with antibiotic treatment for at least 5 days or until death. The management of VLBW neonates with suspected or proven early-onset bacterial sepsis followed the guidelines of the American Academy of Pediatrics [30]. Non-susceptibility was defined as intermediate or resistant according to the final antibiogram.

Small, appropriate, and large for GA were defined as BW below the 10th, within the 10th and 90th, and above the 90th percentile, respectively, using Fenton 2013. Prolonged pre-labor rupture of membranes was defined as the rupture of amniotic membranes more than 18 h before preterm delivery. Clinical chorioamnionitis is typically based on the presence of maternal fever of greater than 38 °C and at least two of the following criteria: maternal leukocytosis, maternal tachycardia, fetal tachycardia, uterine tenderness, and/or foul odor of the amniotic fluid [55].

The diagnosis of ventilator-associated pneumonia and central line-associated bloodstream infection was based on the recent National Healthcare Safety Network guidelines for infants aged <1 year [34,56]. Necrotizing enterocolitis was defined according to the modified Bell’s classification as definite or advanced (stage II or III) [57]. Bronchopulmonary dysplasia was defined as oxygen requirement at 36 weeks’ postmenstrual age for infants born before 32 weeks of gestation and was classified as mild, moderate, or severe based on respiratory support requirements according to the National Institute of Health consensus definitions [58]. The mortality rate at discharge was defined as in-hospital mortality. ENM was defined as mortality within 7 days of life. Length of hospital stay was defined as the duration of hospital stay during admission until discharge or death.

### 4.3. Statistical Analysis

The R program (version 4.5.1; R Foundation for Statistical Computing, Vienna, Austria [59]), including the EpiCalc package (version 4.1.0.1; Epidemiology Unit, Prince of Songkla University, Songkhla, Thailand [60]) was used for data analysis. Categorical variables are presented as frequency and percentage and were compared using the χ^2^ test or Fisher’s exact test. Continuous variables were assessed for normality using the Shapiro–Wilk test. Non-normally distributed continuous variables are presented as medians (IQR and/or range) and were compared using the Mann–Whitney U test (Rank-sum test).

Univariable and multivariable analyses were performed. Variables with *p*-values < 0.2 in the univariable analysis, along with those having biological plausibility, were included in a multivariable backward stepwise logistic regression model. Adjusted OR and 95% CI were computed for variables that were independently associated with EOS versus non-EOS, and ENM versus non-ENM. Outcomes associated with EOS were also analyzed. All *p*-values were two-tailed, and *p* < 0.05 was considered statistically significant.

During the preparation of this manuscript, the authors used Notebook LM to generate an AI infographic for the graphical abstract. The authors have reviewed and edited the output and take full responsibility for the content of this publication.

## 5. Conclusions

In Thailand, the incidence of EOS among VLBW infants was 4.0%, with *E. coli* and *A. baumannii* identified as the predominant pathogens. Clinical chorioamnionitis and 5 min Apgar score < 7 were established as significant independent risk factors for EOS, and infection was strongly associated with increased ENM. Notably, *A. baumannii* exhibited extensive antimicrobial resistance, representing a distinct microbiological profile that is in stark contrast to the GBS-dominant patterns observed in the USA, Europe, and Israel. These findings underscore the critical shift in neonatal epidemiology in Southeast Asia, where environmental and multidrug-resistant Gram-negative bacteria increasingly complicate EOS. Further research on multimodal interventions and specific risk factors for CRAB colonization is urgently needed to mitigate antimicrobial resistance and enhance outcomes in this vulnerable preterm population.

## Figures and Tables

**Table 1:** Comparison of maternal and neonatal baseline data between early-onset sepsis (EOS) and non-EOS groups.

Baseline Characteristics	EOS (*n* = 32)	Non-EOS (*n* = 760)	*p*-Value
Gestational age, weeks, median (interquartile range)	27 (26–28)	28 (26–29)	0.04
Birth weight, g, median (interquartile range)	845 (695–1084)	965 (783–1191)	0.03
Birth weight for gestational age, *n* (%)			>0.99
appropriate for gestational age	27 (84.4)	629 (82.8)	
small for gestational age	5 (15.6)	126 (16.6)	
large for gestational age	0 (0)	5 (0.7)	
Type of hospital, *n* (%)			0.02
university-affiliated hospitals	18 (56.2)	357 (47.0)	
public hospitals	14 (43.8)	403 (53.0)	
Male sex, *n* (%)	17 (53.1)	421 (55.4)	0.94
Prolonged prelabor rupture of membranes, *n* (%)	6 (18.8)	117 (15.4)	0.62
Clinical chorioamnionitis, *n* (%)	9 (28.1)	50 (6.6)	<0.001
Maternal urinary tract infections, *n* (%)	0 (0)	17 (2.2)	>0.99
Use of prophylactic antibiotics in labor and delivery, *n* (%)	21 (65.6)	404 (53.2)	0.23
Duration of antibiotic prophylaxis more than 4 h before delivery, *n* (%)	12 (37.5)	231 (30.4)	0.51
Antenatal corticosteroid, *n* (%)	27 (84.4)	549 (72.2)	0.19
Antenatal magnesium sulfate, *n* (%)	14 (43.8)	208 (27.4)	0.07
Multifetal gestations, *n* (%)	5 (15.6)	114 (15.0)	0.81
Vaginal delivery, *n* (%)	19 (59.4)	362 (47.6)	0.26
Inborn, *n* (%)	30 (93.8)	640 (84.2)	0.21
5 min Apgar score, median (interquartile range)	6 (4–8)	7 (6–9)	0.007
5 min Apgar score less than 7, *n* (%)	20 (62.5)	264 (34.7)	0.003

**Table 2:** Comparison of clinical outcomes and neonatal morbidities during hospitalization between early-onset sepsis (EOS) and non-EOS groups.

Neonatal Data	EOS (*n* = 32)	Non-EOS (*n* = 760)	*p*-Value
Ventilator-associated pneumonia, *n* (%)	6 (18.8)	285 (37.5)	0.07
Central line-associated bloodstream infection, *n* (%)	0 (0)	56 (7.4)	0.27
Necrotizing enterocolitis, *n* (%)	0 (0)	19 (2.5)	>0.99
Bronchopulmonary dysplasia, *n* (%)	0 (0)	11 (1.4)	>0.99
Persistent pulmonary hypertension of the newborn, *n* (%)	2 (6.2)	14 (1.8)	0.13
Mortality rate at discharge, *n* (%)	13 (40.6)	133 (17.5)	0.002
Early neonatal mortality rate, *n* (%)	10 (31.2)	39 (5.1)	<0.001
Length of hospital stay (total cases), days *	59 (5–86)	76 (52–109)	0.02
Length of hospital stay (only survivors), days *	80 (70–138)	86 (63–114)	0.66

* Data are presented as median (interquartile range).

**Table 3:** Antimicrobial non-susceptibility profiles of causative pathogens isolated from neonates with early-onset sepsis.

Pathogens	Deaths, *n* (%)	Aminoglycosides, *n* (%)		Cefotaxime, *n* (%)	Meropenem, *n* (%)	Methicillin, *n* (%)
		Amikacin	Gentamicin			
*Escherichia coli*	3/10 (30.0%)	0/10 (0)	4/9 (44.4)	3/8 (37.5)	0/9 (0)	NA
*Acinetobacter baumannii*	5/9 (55.6%)	4/6 (66.7)	5/6 (83.3)	3/4 (75.0)	5/6 (83.3)	NA
Coagulase-negative staphylococci or *Staphylococcus epidermidis*	2/7 (28.6%)	NA	5/5 (100)	NA	NA	5/5 (100)
*Campylobacter fetus*	0/1 (0%)	NA	NA	NA	NA	NA
*H* *aemophilus influenzae*	2/2 (100%)	NA	NA	0/2 (0)	0/2 (0)	NA
*Klebsiella pneumoniae*	1/1 (100%)	1/1 (100)	1/1 (100)	1/1 (100)	1/1 (100)	NA
*Listeria monocytogenes*	0/1 (0%)	NA	NA	NA	NA	NA
*Micrococcus species*	0/1 (0%)	NA	NA	NA	NA	NA
*Moraxella species*	0/1 (0%)	NA	NA	NA	NA	NA
*Pseudomonas aeruginosa*	1/1 (100%)	0/1 (0)	0/1 (0)	1/1 (100)	NA	NA
*Staphylococcus aureus*	0/1 (0%)	NA	NA	NA	NA	0/1 (0)
*Staphylococcus capitis*	1/1 (100%)	NA	NA	NA	NA	1/1 (100)
*Staphylococcus hominis*	0/1 (0%)	NA	NA	NA	NA	0/1 (0)
*Streptococcus agalactiae*	0/1 (0%)	NA	NA	NA	NA	NA
Total		5/18 (27.8)	15/22 (68.2)	8/16 (50.0)	6/18 (33.3)	6/8 (75.0)

NA, not available.

**Table 4:** Factors associated with early-onset sepsis: Univariable and multivariable analysis.

Risk Factors	Univariable Analysis		Multivariable Analysis	
	Odds Ratio (95% Confidence Interval)	*p*-Value	Adjusted Odds Ratio (95% Confidence Interval)	*p*-Value
Gestational age	0.99 (0.98–0.99)	0.04	-	
Prolonged prelabor rupture of membranes	1.27 (0.51–3.15)	0.62	-	
Clinical chorioamnionitis	5.56 (2.44–12.64)	<0.001	1.12 (1.07–1.18)	<0.001
Antenatal corticosteroid	2.08 (0.79–5.46)	0.16	-	
Antenatal magnesium sulfate	2.06 (1.01–4.23)	0.07	1.03 (0.99–1.06)	0.05
5 min Apgar score less than 7	3.13 (1.51–6.51)	0.002	1.05 (1.02–1.08)	0.002

**Table 5:** Factors associated with early neonatal mortality (ENM) in very preterm infants.

Baseline Characteristics	ENM (*n* = 49)	Non-ENM (*n* = 743)	*p*-Value
Gestational age, weeks, median (interquartile range)	26 (24–28)	28 (26–29)	<0.001
Birth weight, g, median (interquartile range)	800 (614–1055)	970 (790–1199)	<0.001
Birth weight compared to gestational age, *n* (%)			0.14
appropriate for gestational age	36 (73.5)	620 (83.4)	
small for gestational age	13 (26.5)	118 (15.9)	
large for gestational age	0 (0)	5 (0.7)	
Type of hospital			0.02
university-affiliated hospitals	15 (30.6)	360 (48.5)	
public hospitals	34 (69.4)	383 (51.5)	
Male sex, *n* (%)	29 (59.2)	409 (55.0)	0.68
Prolonged prelabor rupture of membranes, *n* (%)	9 (18.4)	114 (15.3)	0.72
Clinical chorioamnionitis, *n* (%)	5 (10.2)	54 (7.3)	0.40
Maternal urinary tract infections, *n* (%)	1 (2.0)	16 (2.2)	>0.99
Use of prophylactic antibiotics in labor and delivery, *n* (%)	21 (65.6)	404 (53.2)	0.23
Duration of antibiotic prophylaxis more than 4 h before delivery, *n* (%)	12 (24.5)	231 (31.1)	0.42
Antenatal corticosteroid, *n* (%)	33 (67.3)	543 (73.1)	0.48
Antenatal magnesium sulfate, *n* (%)	11 (22.4)	211 (28.4)	0.46
Multifetal gestations, *n* (%)	10 (20.4)	109 (14.7)	0.38
Vaginal delivery, *n* (%)	27 (55.1)	354 (47.6)	0.39
Inborn, *n* (%)	30 (93.8)	640 (84.2)	0.21
5 min Apgar score, median (interquartile range)	6 (5–7)	7 (6–9)	<0.001
5 min Apgar score less than 7, *n* (%)	30 (61.2)	254 (34.2)	<0.001
Comorbidities during the first days of life, *n* (%)			
Early-onset sepsis	10 (20.4)	22 (3.0)	<0.001
Respiratory distress syndrome	8 (16.3)	14 (1.9)	<0.001
Persistent pulmonary hypertension of the newborn	9 (18.4)	7 (0.9)	<0.001

**Table 6:** Univariable and multivariable analyses of factors associated with early neonatal mortality.

Risk Factors	Univariable Analysis		Multivariable Analysis	
	Odds Ratio (95% Confidence Interval)	*p*-Value	Adjusted Odds Ratio (95% Confidence Interval)	*p*-Value
Early-onset sepsis	8.40 (3.72–18.97)	0.001	1.26 (1.16–1.36)	<0.001
Gestational age	0.98 (0.97–0.99)	<0.001	0.98 (0.97–0.99)	<0.001
Birth weight	0.99 (0.99–0.99)	<0.001		
Public hospitals	1.18 (0.66–2.13)	0.66	1.06 (1.03–1.09)	<0.001
Birth weight compared to gestational age, (appropriate for gestational age = reference)	1		1	
small for gestational age	1.05 (0.99–1.09)	0.05	1.06 (1.02–1.11)	0.007
large for gestational age	0.95 (0.77–1.17)	0.61	0.94 (0.77–1.14)	0.54
5 min Apgar score < 7	3.04 (1.68–5.51)	<0.001		
Persistent pulmonary hypertension of the newborn	2.30 (1.91–2.76)	<0.001	1.62 (1.46–1.81)	<0.001

## Data Availability

Raw data supporting the conclusions of this study will be provided by the authors upon request. The data are not publicly available due to privacy concerns.

## Abbreviations

The following abbreviations are used in this manuscript:

aOR	adjusted odds ratio
CDC	Centers for Disease Control and Prevention
CoNS	coagulase-negative staphylococci
BW	birth weight
CFR	case fatality rate
CI	confidence interval
CRAB	carbapenem-resistant *A. baumannii*
CSF	cerebrospinal fluid
ENM	early neonatal mortality
EOS	early-onset sepsis
GA	gestational age
GBS	Group B Streptococcus
IAP	intrapartum antibiotic prophylaxis
IQR	interquartile range
NICHD	National Institute of Child Health and Human Development
NICU	neonatal intensive care unit
PPHN	persistent pulmonary hypertension of the newborn
SGA	small for gestational age
ThaiNy	Thai Neonatal Registry
VLBW	very low birth weight
